# Intercultural Dialogue Begins at the Dining Table: A Unilateral Kosovo Perspective on Turkish–Kosovar Fusion Cuisine

**DOI:** 10.3390/foods15020222

**Published:** 2026-01-08

**Authors:** Ceyhun Uçuk, Çağın Çevik, Onurcan Arman, Charles Spence

**Affiliations:** 1 Gastronomy and Culinary Arts Department, Gaziantep University, University Boulevard, Gaziantep 27100, Türkiye; ceyhunucuk@gantep.edu.tr; 2 Faculty of Applied Sciences, Gastronomy and Culinary Arts Department, Istanbul Gelisim University, Avcılar, Istanbul 34310, Türkiye; 3 Department of Biophysics, Cerrahpasa Faculty of Medicine, Istanbul University-Cerrahpasa, Fatih, Istanbul 34098, Türkiye; cagin.cevik@ogr.iuc.edu.tr; 4 Institute for Physical Activity and Nutrition (IPAN), School of Exercise and Nutrition Sciences, Deakin University, Geelong, VIC 3216, Australia; onurcanarman132@gmail.com; 5 School of Psychology, Aston University, Birmingham B4 7ET, UK; 6 Department of Experimental Psychology, Life and Mind Building, South Parks Road, University of Oxford, Oxford OX1 3PS, UK

**Keywords:** fusion cuisine, gastronomy, culture, culinary diplomacy

## Abstract

Fusion cuisine blends ingredients, cooking techniques, and flavours from different cultures, yet little is known about how it is perceived within the context of gastrodiplomacy. This study explores perceptions of fusion cuisine at a multicultural gastrodiplomacy event held in Kosovo, where the participants first sampled Turkish–Kosovar fusion dishes during tasting sessions and subsequently completed an online questionnaire designed to assess their experience. In this event, participants attended structured tasting activities in Prizren and Pristina, where they sampled dishes combining elements of both culinary traditions, and then completed an online structured questionnaire consisting of 5-point Likert-type items evaluating their fusion cuisine preferences. The study was conducted in Kosovo as part of a unilateral gastrodiplomatic initiative. A total of 451 participants responded to an online questionnaire, which included fusion cuisine preference scores and metaphorical descriptions of their culinary experiences. A key contextual characteristic of this study is that data were collected exclusively during a fusion cuisine event held in Kosovo, with participation from a multinational audience who attended the event. Therefore, the sample reflects diverse cultural backgrounds within a single-location setting. The results indicate that younger, highly educated, and higher-income participants exhibited significantly greater openness to culinary diversity. These findings advance the state of knowledge by demonstrating that public reception of gastrodiplomacy is stratified by socioeconomic factors rather than defined solely by national background. Practically, this implies that effective fusion-based diplomacy requires targeted strategies to bridge demographic gaps and ensure broader social inclusivity, rather than relying on a one-size-fits-all approach.

## 1. Introduction

Fusion cuisine is an innovative gastronomic approach resulting from the bringing together of ingredients, cooking techniques, and/or flavours from different cultures, and nowadays, it attracts great attention worldwide (see [1] for a review). The approach offers consumers experiences that are both familiar and unique by consciously synthesizing the materials, techniques, and/or esthetics of different cultures. Those working in the world of fusion cuisine have helped to introduce unusual flavour experiences, not only as a combination of traditional elements, but also as a product of interdisciplinary creativity, cultural interaction, and the emergence of innovative gastronomic movements [1,2,3,4]. Fusion cuisine is often understood as a deliberate and intentional blending of culinary traditions. Yet its foundations also lie in the (more unplanned) integration of regional cuisines that have occurred as a result of imperial histories and colonial encounters. Dishes such as curry and chicken tikka masala exemplify this latter process of cultural exchange, which in turn provided the groundwork for contemporary notions of fusion cuisine [5]. Consequently, the development of a novel fusion meal nowadays frequently signifies not an entirely original mixture, but rather a creative reimagining of established historical knowledge (see [1] for a review).

However, despite the extensive global literature on fusion cuisine, no empirical research has to date examined fusion cuisine specifically between Türkiye and Kosovo, even though these two culinary cultures share deep-rooted historical ties through Ottoman gastronomic heritage. This absence constitutes an important research gap, as the Balkans represent one of the regions where Turkish and local food traditions have intersected for centuries [6,7]. Yet the contemporary hybridization of these cuisines—especially the way in which Kosovar consumers perceive Turkish–Kosovar fusion dishes—remains unexplored.

Cultural interactions, migration, and globalization have fostered the development of novel flavour combinations/experiences and methods in gastronomy [8,9]. This heightens consumers’ enthusiasm for culinary discoveries and cultivates a desire to sample dishes made using ingredients from diverse civilizations [1]. Stano [10] underscores the fact that such synthesis may not consistently produce harmonious outcomes; at times, incompatible amalgamations of diverse flavours and textures can also result in confusion, sometimes termed ‘con-fusion cuisines’. In Malaysia, for example, fusion cuisine has been shaped by the combination of local, Chinese, English colonial rule and Indian cuisines, reflecting the multicultural structure of the country. This diversity ensures both the preservation of cultural heritage as well as the development of innovative dishes [11,12].

The development of fusion cuisine is not only related to the combination of flavours and cooking techniques but also to the innovation of esthetic presentation and gastronomic experience [13,14,15]. Consumers with a higher level of education generally tend to be more interested in new tastes and gastronomic experiences [16]. Spence [1] emphasizes that educated consumers tend to appreciate gastronomic innovations resulting from the combination of different cultural elements, and these individuals are often more open to innovative cooking techniques. Fusion cuisine is not an arbitrary amalgamation of visually incongruous elements; rather, it is a deliberate cultural and technical synthesis that seeks to modify culinary boundaries [17,18,19].

Spence [1] highlights that numerous contemporary dishes are, in fact, amalgamations of ingredients, flavours, techniques, and even philosophical concepts. Fusion food has been popularized in the contemporary era by trailblazers such as Wolfgang Puck [20]. This culinary style has served as a synthesis of technological methods and a manifestation of media-centric, chef-oriented gourmet discourses. Moreover, it is known that young consumers have easier access to different cuisines through social media and digital platforms and are therefore more open to innovative tastes [21]. Kovalenko et al. [22] revealed that young consumers are more interested in esthetic and creative presentations and are more open-minded to such gastronomic experiences than older generations. In contrast, older consumers tend to stick to more traditional tastes and food preparation techniques. Therefore, the appeal of fusion cuisine may be more pronounced, especially among young consumers.

As a result of globalization, the rapidly evolving field of gastronomy has seen the emergence of fusion cuisine as a powerful symbol of new tastes and cultural encounters. However, the impact of such gastronomic preferences on intercultural understanding and diplomatic rapprochement has received little attention to date. Therefore, the purpose of the present study was to gain a better understanding of fusion cuisine’s potential as an instrument of gastrodiplomacy.

The literature on gastrodiplomacy has yet to incorporate empirical evidence from hybrid cuisines situated within the Anatolian-Balkan cultural corridor. Unlike the commonly studied Asian-Western or Afro-European fusion cuisines, the Türkiye–Kosova fusion context represents a historically intertwined yet academically largely overlooked domain [23,24,25,26]. This creates an opportunity to examine the diplomatic implications of shared culinary heritage between two culturally intertwined nations.

Fusion cuisine, which combines various culinary traditions and ingredients, has made significant contributions to modern gastronomy and gastrodiplomacy, reflecting both globalization and cultural change. Numerous studies emphasize the way in which fusion cuisine serves as a tool for cultural connection [1,27]. When done well, fusion cuisine successfully preserves different cultural identities in a single dish [28].

Fusion cuisine can also be shaped by migration, as immigrant communities often play an influential role in the emergence of new hybrid dishes [29]. Świtała-Trybek [30] has argued that traditional dishes have become more modern by incorporating foreign culinary elements into them. Fusion cuisine also supports culinary tourism by contributing to the gastronomic appeal of the regions [30]. Globalization has undoubtedly facilitated the spread of fusion cuisine. In fact, some commentators claim that fusion cuisine offers benefits, such as promoting cultural diplomacy, and challenges, such as debates concerning authenticity [31,32].

Srivastav et al. [33] demonstratethat the success behind the appreciation of fusion cuisine by local people in places such as India is attributable to the blending of local flavours with global techniques that cater to different consumer preferences. Dimoula et al. [34] emphasize the historical depth of such practices, emphasizing that the Mediterranean region has long been a hub for culinary fusion. Rodrigues [35] considers Macau cuisine, which combines Chinese and Portuguese elements, as a longstanding example of culinary fusion that goes beyond food, symbolizing the region’s cultural resilience and adaptability.

Fusion cuisine can be considered a culinary expression of cross-cultural adaptation, a way in which individuals or groups adapt to new cultural environments [36]. According to cross-cultural adaptation theory [36], adaptation occurs when people encounter unfamiliar cultural environments and gradually come to integrate aspects of the new culture with elements of their original cultural framework. Fusion cuisine reflects this adaptive process. Chefs and communities draw from multiple culinary traditions and selectively blend flavours, ingredients, and cooking methods as they navigate intercultural encounters. Fusion cuisine therefore represents an adaptive interplay between cultural heritage and new cultural influences, rather than solely mixing cuisines for novelty [1].

Fusion cuisine also plays a critical role in the context of gastrodiplomacy, where gastronomic elements may be used as a tool to help build bridges (and mutual understanding) between cultures. Public diplomacy that uses a country’s culinary culture to strengthen interpersonal relations and create a positive image in the international arena is called gastrodiplomacy or culinary diplomacy [37,38]. Rockower [39] emphasizes how nations use fusion food as a ‘soft power’ tool in order to improve international relations by bridging cultural gaps, by presenting their cultures in dynamic, accessible ways and by blending local and foreign traditions. Including fusion culinary practices in gastrodiplomacy activities can help to make gastrodiplomacy a more effective tool. Moreover, gastrodiplomacy also aligns with Nye’s concept of soft power [40], defined as the ability to have preferred outcomes through attraction and cultural appeal rather than the use of coercive force. Food operates as a form of soft power as it can create positive emotional responses and help to shape perceptions of a country or culture [41]. When governments or cultural groups promote their national cuisine abroad, such as through food festivals or cultural programmes, they are, in a sense, engaging in gastrodiplomacy. They aim to enhance their culture’s image and strengthen international relationships. By using food as a cultural tool, gastrodiplomacy allows people to experience a nation’s values and identity in a way that is easy to access and appealing [42].

Nowadays, individuals with a higher level of education generally have a broader cultural background and exhibit greater openness to diverse culinary experiences [18]. These individuals tend to be more curious about innovative tastes and cooking techniques, which increases their interest in foods that blend different cultures, such as fusion cuisine [16]. In particular, the combination of ingredients and cooking techniques from diverse cultures has been perceived as a form of gastronomic innovation and has been appreciated more by educated consumers. Indeed, individuals with a higher level of education value gastronomic diversity and innovations more, while those with a lower level of education are more likely to prefer traditional tastes [43]. The level of education stands out as a determining factor in the rate of preference for foods that blend different cultural elements. In this context, the following hypothesis has been developed:

**H1:**  *Individuals with higher levels of education will demonstrate an increased preference for foods and beverages incorporating ingredients from diverse culinary traditions compared to those with lower levels of education.*

On the one hand, young consumers have easier access to visual descriptions of world cuisines thanks to digital media and social networks and have developed an open-mindedness towards trying new flavours [21]. This group shows great interest in gastronomic innovations and esthetic presentations, especially those that happen to be trending on social media [22]. On the other hand, older consumers tend to stick to more traditional tastes and food preparation techniques, which may limit their interest in different culinary cultures. Many studies have confirmed that young consumers are more sensitive to esthetic and creative presentations and are more open to such gastronomic experiences [28]. Therefore, recipes that amalgamate ingredients, techniques, components, and even philosophical perspectives from different cultures are more appealing to young customers [1,35]. Moreover, young consumers are more interested in food experiences that combine cooking techniques from different nations and/or cultures as compared to older consumers. In this context, the following hypothesis is proposed:

**H2:**  *Dishes that combine cooking techniques from different nations (food cultures) are more appealing to young consumers than to those who are older.*

Individuals with higher income levels can generally allocate more of their disposable income to food and beverage experiences from different culinary cultures. This consumer group is also interested in luxurious and unique gastronomic experiences and prefers creative and esthetic presentations [16]. Unique flavours/flavour combinations that are created from the combination of different culinary cultures are appreciated more, especially through creative presentations. High-income consumers consider that esthetic presentations contribute to the overall experience of the meal and give more positive responses if these presentations happen to be both innovative and creative [44]. Moreover, gastronomic experiences are not only limited to taste, but plating and presentation styles are also among the determining factors in consumer preferences [45,46]. In this context, based on the result that creative presentations increase the interest of high-income individuals in fusion foods and beverages, the following hypothesis was developed:

**H3:**  *High-income individuals are more interested in fusion foods and beverages from diverse culinary cultures.*

The obligation to comply with halal food rules is an important factor in the food preferences of Muslim consumers, which may lead them to be more cautious towards fusion cuisine products whose provenance may be unknown [47]. Foods that do not have a halal certificate or are prepared with non-halal cooking techniques and ingredients may not be considered trustworthy by Muslim consumers. In contrast, Christian or other religious consumers may be more flexible in their food preferences and may be more likely to consume foods resulting from the combination of different culinary cultures due to their having fewer religious restrictions [48]. As a result, they may be more interested in new and innovative flavours. For example, Shafie and Othman [49] state that halal food safety and perception are important issues for Muslims, and therefore, they may be less likely to consume products of diverse cultural cuisines. Since Muslim consumers may be more cautious towards unique foods resulting from the combination of different culinary cultures due to religious restrictions, the following hypothesis was developed:

**H4:**  *Muslim consumers might be less likely to consume products that combine elements of different culinary cultures than consumers of other religious backgrounds.*

Nationality significantly shapes consumers’ food choices, particularly through cultural traditions and exposure to specific culinary practices. Kosovo has been under the influence of the Ottoman Empire and other civilizations throughout recorded history, which has led to the adoption of elements belonging to different nations in their culinary culture [50]. This diversity may be the reason why Kosovan consumers are more open to trying foods from different cultures. In contrast, some countries in the Balkans may have a more homogeneous culinary culture, and consumers in these countries may be more likely to stick to those dishes that can be considered traditional [51]. This situation leads to the conclusion that the multicultural structure and food diversity in Kosovo are greater than in other Balkan countries and that consumers may have a more positive view of such culinary experiences [52,53]. In this context, the following hypothesis was developed:

**H5:**  *Kosovan consumers are more likely to taste flavours prepared with ingredients from different nationalities than consumers from other Balkan countries.*

Those with a higher level of education are more open to gastronomic experiences and appreciate the cultural and historical context behind these experiences more [1]. Educated consumers are more willing to try new flavours and are more interested in creative and innovative dishes that blend elements from different cuisines. In contrast, individuals with lower levels of education generally tend to prefer more familiar and traditional foods and may be more cautious about fusion cuisine [54,55]. The trend towards fusion cuisine varies by income and education level: For example, while high-income individuals show more interest in creative and esthetic food presentations, those individuals with higher levels of education tend to appreciate different cultural elements more [56,57]. In this context, the hypothesis developed is as follows:

**H6:**  *In individuals with higher levels of education, the level of interest in fusion dishes and beverages that combine ingredients from different culinary cultures increases significantly as their income level increases.*

Therefore, the purpose of the present study is to gain a greater understanding of fusion cuisine’s potential as an instrument of gastrodiplomacy within the specific context of Türkiye and Kosovo. By addressing the identified gap in the literature, this research aims to empirically test how demographic variables—specifically education, age, income, religion, and nationality—influence the perception and preference for fusion dishes that bridge these two historically intertwined cultures.

## 2. Materials and Methods

This study used a quantitative research design to explore the factors influencing preferences for fusion cuisine across diverse demographic groups.

### 2.1. Participants

The data for this study were gathered as part of a gastrodiplomacy initiative between Kosovo and Türkiye, designed to promote cultural exchange via fusion cuisine. The project encompassed fusion cuisine tasting events conducted in Prizren and Pristina, allowing participants to sample dishes (see Appendix B) that amalgamated aspects of both Turkish and Kosovar culinary traditions. After the tasting sessions, the participants were requested to fill out a structured questionnaire designed to assess their perceptions and preferences concerning fusion cuisine. The questionnaire comprised Likert-scale items gastronomic experiences. A total of 451 people took part in the study. Participation was optional and confidential, with all respondents giving their informed consent prior to completing the survey.

The distribution of participants by nationality was as follows: 17.3% (n = 78) from Kosovo, 15.5% (n = 70) from Albania, 13.3% (n = 60) from Serbia, 12.4% (n = 56) from North Macedonia, 9.3% (n = 42) from Montenegro, 7.8% (n = 35) from Romania, 7.1% (n = 32) from Bulgaria, 6.6% (n = 30) from Bosnia and Herzegovina, 6.2% (n = 28) from Croatia, and 4.4% (n = 20) from other countries.

The gender distribution was 45% female (n = 203) and 55% male (n = 248). The participants were grouped into the following age categories: 18–29 years (23.3%, n = 105), 30–39 years (24%, n = 108), 40–49 years (18.2%, n = 82), 50–59 years (18.8%, n = 85), and 60 years and above (15.7%, n = 71). Additionally, participants were classified based on their marital status, religion, educational attainment, and income levels. The detailed distribution of participants across these educational and income categories, which are relevant to Hypotheses H1, H3, and H6, is presented in Table 1.

To assess demographic comparability across national groups, Chi-square tests were conducted for both gender and age-group distributions. The results indicated no statistically significant differences in gender (χ^2^ = 4.72, df = 9, *p* = 0.78) or age-group distributions (χ^2^ = 11.56, df = 36, *p* = 0.99) across nationalities, suggesting comparable distributions. A priori ANOVA power (G*Power 3.1; f = 0.25, α = 0.05, 1 − β = 0.80) implies ≈53/group for k = 3 or ≈45/group for k = 4. Because the H5 analysis used four nationality groupings (Kosovo, Croatia, North Macedonia, Other countries) and some cells were small (n = 28), H5 is treated as underpowered/exploratory, and effect sizes are reported alongside *p* values (Table 2).

To accommodate linguistic preferences, the participants were divided into four language groups: Turkish (26.2%, n = 118), Kosovan (60.5%, n = 273), Albanian (10.9%, n = 49), and English (2.4%, n = 11). Since both Albanian and Turkish are widely spoken native languages in Kosovo—depending on the region—the data were collected in three languages (Albanian, Turkish and English) to ensure accurate comprehension and response validity.

To minimize potential self-selection bias, participant recruitment was conducted in person during two structured gastrodiplomacy tasting events in Prizren and Pristina, where all of the attendees were invited to participate regardless of demographic background or culinary interest. The survey link was provided immediately after the tasting session to all participants under identical conditions, and no incentives were offered to avoid attracting only highly motivated individuals. Participation rates were documented automatically through the survey platform, allowing for verification that the final sample closely reflected the demographic distribution of event attendees, thereby reducing systematic self-selection effects.

### 2.2. Context of the Event and Execution of the Survey

The fusion cuisine tasting events were conducted on [3 August 2024–4 August 2024] in Prizren and Pristina as a component of the gastrodiplomacy initiative. The participants were presented with a carefully prepared assortment of fusion dishes that combined Kosovar and Turkish ingredients and culinary techniques. Immediately following the tasting session, the participants were prompted to complete a survey on an internet platform, facilitating prompt recollection of their sensory experiences. The poll was offered in Turkish, Albanian, and English, enabling the respondents to articulate their opinions in their preferred language. The criteria for establishing fusion cuisine included the preservation of traditional culinary features and the assessment of geographical connections. The following fundamental aspects were considered in the dish selection process:

- Kosovo and Turkish cuisines have historical gastronomic roots, including influences from Ottoman cuisine and Mediterranean dietary practices. Fundamental ingredients, such as spices (cumin, sumac, coriander powder), grains products (bulgur as a whole grain; cornflour as a refined grain product), meats (lamb, beef), dairy (yoghurt, white cheese) and legumes (chickpeas, lentils), common to both cuisines, were emphasized in the meal selection process. Novel flavours emerged from the fusion of roasting and stewing techniques prevalent in Turkish cuisine when combined with the baking and prolonged cooking procedures characteristic of Kosovan cuisine.
- By means of research, the taste profiles (sweet, sour, umami, bitter, salty) were harmonized, and cross-flavour combinations that were designed to appeal to both Kosovo and Turkish cultures were developed.

Although the event was organized collaboratively by institutions from Turkey and Kosovo, participant recruitment occurred solely in Kosovo due to practical limitations and ethical approval (which was limited to individuals in Kosovo, thus precluding the inclusion of Turkish volunteers). Consequently, instead of performing a pairwise comparison with Turkish participants, the study examines the evaluation of Turkish–Kosovo fusion foods by various Kosovar audiences during the gastrodiplomacy programme.

### 2.3. Data Collection

The multi-item constructs included in the questionnaire were adapted from the Fusion Cuisine Eating Tendency dimension of a validated psychometric scale, conceptualizing fusion cuisine preference as a psychological disposition related to curiosity, openness, and willingness to explore culturally hybrid food experiences [18,54]. This measurement approach aligns conceptually with theories such as Sensation Seeking and Food Neophobia, which describe individuals’ motivational and affective reactions toward novel food stimuli. Accordingly, the study does not rely solely on demographic associations but incorporates a theory-driven behavioural component, enabling deeper interpretation of consumer responses to fusion cuisine. The adaptation process included expert review and wording adjustments to reflect the Türkiye-Kosova fusion context while preserving the original construct structure. The scale demonstrated high internal consistency in the present study (Cronbach’s α = 0.86), confirming reliable measurement. The full list of questionnaire items used to assess openness, preferences, and gastrodiplomacy perceptions remains accessible in Appendix A, ensuring transparency and enabling replication. The Albanian translation of the questionnaire presented in Appendix A was carried out and linguistically validated by a professional translator from the Yunus Emre Institute in Kosovo, ensuring accurate cultural and semantic equivalence between the Turkish and Albanian versions. Additionally, Appendix B provides the standardized recipes of the fusion dishes that were served during the tasting activity, further strengthening systematic reproducibility.

### 2.4. Data Analysis

In this study, the validity of the sample sizes and the appropriateness of the statistical analysis methods were carefully evaluated to ensure the reliability of the hypothesis testing process. According to a priori power analysis (G*Power 3.1), for one-way analyses of variance (ANOVAs) with a medium effect (Cohen’s f = 0.25), α = 0.05, and 1 − β = 0.80, the required total sample is N ≈ 159 for k = 3 (≈53 per group) and N ≈ 180 for k = 4 (≈45 per group). For two-group comparisons (H6), an independent-samples t test with Cohen’s d = 0.50 requires ≈64 per group.

In this context, f represents the standardized effect size used in power estimation, alpha (α) denotes the Type I error probability, and beta (β) represents the Type II error rate corresponding to 80% statistical power. The power analysis confirmed that the final dataset exceeded the recommended sample thresholds, ensuring adequate sensitivity to detect medium-sized differences across groups.

Although the total sample size met conventional thresholds for parametric testing, nonparametric statistical methods were preferred because 5-point Likert-type items constitute ordinal-level data. In accordance with established methodological recommendations, ordinal scales do not assume equal intervals between categories and therefore are more appropriately analyzed using distribution-free tests such as the Kruskal–Wallis and Mann–Whitney U tests [58,59]. Hence, the selection of statistical techniques in this study was based on the measurement properties of the data rather than sample size.

Accordingly, Kruskal–Wallis tests were used to assess group differences for H1 (education), H2 (age), H3 (income), H4 (religion), and H5 (nationality). When significant effects were identified, Dunn’s post hoc tests with Bonferroni correction were applied. Hypothesis H6, which examined income differences among highly educated participants, was analyzed using the Mann–Whitney U test. Effect sizes were calculated using ordinal eta-squared (η^2^_o_) for Kruskal–Wallis tests and Cliff’s delta (δ) for Mann–Whitney U tests. A significance threshold of *p* < 0.05 was applied throughout.

The design of this study was strictly correlational, focusing on group differences as specified by the hypotheses (H1–H6); hence, complex analyses such as regression analysis or factor analysis were not used, as the primary goal was descriptive group comparison rather than predictive modelling. We assessed the comparability of the main groups based on nationality by running Chi-square tests on gender and age distributions in Section 2.1, confirming that these potential demographic factors did not differ significantly across national groups.

Missing data were evaluated for all variables. Cases with less than 5% of missing data were imputed using mean substitution, ensuring minimal bias in the dataset. Variables with more than 5% missing data were excluded from further analyses to maintain statistical validity. Outliers were identified visually through boxplots and statistically using the interquartile range (IQR) method. The extreme 1% of values from both tails of the distribution were trimmed to minimize bias (this procedure remained valid under the nonparametric framework). For visual comparison of group differences, boxplots were annotated using the compact letter display (CLD) method, in which groups sharing the same letter do not differ significantly.

Quantitative data collected from the structured questionnaire were analyzed using IBM SPSS Statistics (Version 20.0). Boxplots were used to visualize the distribution of preference scores across demographic groups, showing median values and interquartile ranges. All visualizations were created using Python (v3.9), specifically using the matplotlib and seaborn libraries for precise and publication-ready graphical representations. This approach was adopted to ensure higher resolution, greater customisation, and adherence to the refined esthetic standards required for peer-reviewed journal publication, as compared to standard statistical software output. Box = IQR (Interquartile Range), centre line = median; whiskers represent the sample minimum and maximum, and no outlier points were drawn. As Likert items are bounded (1–5), this choice emphasizes the full observed range of participants’ responses. The same rule was applied consistently across all figures.

**Figure 1:**
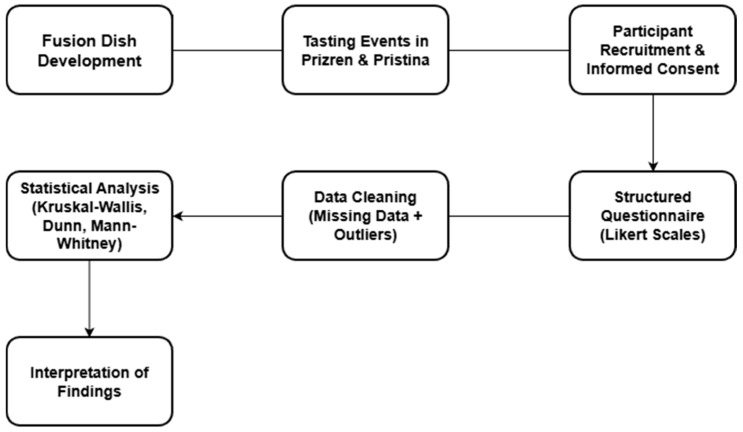
An overview of the methodological workflow. This flowchart summarizes the sequential stages of the research process, beginning with the development of fusion dishes and continuing through participant recruitment, informed consent procedures, and the administration of a structured Likert-scale questionnaire. Subsequent steps include data cleaning (addressing missing values and outliers), statistical analyses, and the final interpretation of findings. The diagram provides a visual representation of the study’s methodological structure and ensures transparency in how each stage contributes to the overall analytical framework (Figure 1).

The methodological practices, such as hypothesis testing, meticulous management of missing and outlier data, and the application of proven statistical methods, are expected to enhance the robustness, reliability, and reproducibility of the study’s results. Chi-square tests were also performed to verify demographic balance amongst national groups based on gender and age distributions. The overall workflow of the research process is summarized visually in the methodological flowchart presented in Figure 1.

## 3. Results

### 3.1. Education Level and Fusion Cuisine Preference

**Figure 2:**
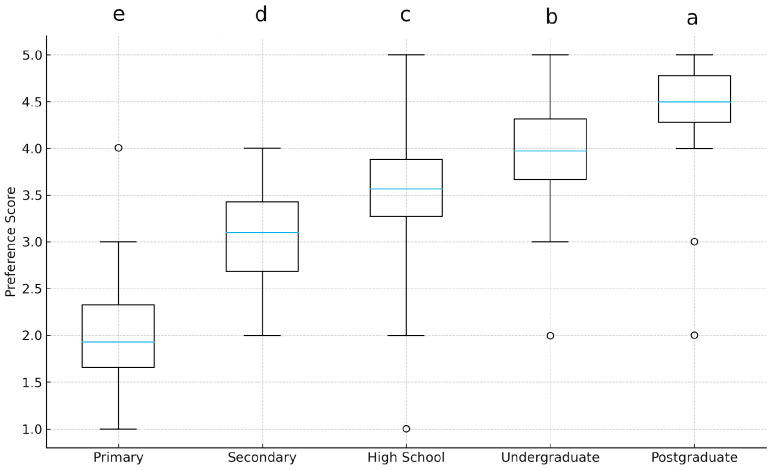
The distribution of preference scores across education levels. Medians are shown as horizontal lines inside the boxes; IQR is indicated by the box boundaries, and whiskers extend to the observed minimum and maximum values (Preference scores (measured on a 1–5 Likert scale, with 1 = strongly disagree and 5 = strongly agree) increased progressively with higher education levels). Different letters above the boxplots indicate statistically significant differences among education groups according to Dunn’s post hoc test (*p* < 0.05).

A Kruskal–Wallis test was conducted to evaluate whether preference scores for combining ingredients from different culinary cultures varied significantly across education levels. The results revealed a statistically significant difference among the groups (H = 32.41, *p* < 0.001, η^2^_o_ = 0.11). As shown in Figure 2, preference scores increased progressively with higher education levels. Participants with postgraduate degrees demonstrated the highest median preference score (median ≈ 4.6), whereas those with primary education exhibited the lowest (median ≈ 1.6).

Dunn’s post hoc analysis (Bonferroni-adjusted) indicated that Postgraduate > Bachelor > Associate > Secondary > Primary (all *p* < 0.05).

These findings offer strong empirical support for Hypothesis 1 (H1), confirming that those individuals with higher educational attainment exhibit significantly greater interest in fusion cuisine than those with less education. The progression of scores underscores the influence of education on culinary openness and the appreciation of gastronomic diversity. Education was associated with fusion-preference, H(4) = 32.41, *p* < 0.001, η^2^_o_ = 0.11 (see Figure 2). The robust statistical significance (*p* < 0.001) suggests that education is a reliable differentiator of preference, yet the small-to-moderate effect size (η^2^_o_ = 0.11) indicates that it accounts for a modest proportion of the overall variance in openness to fusion cuisine (see Figure 2).

### 3.2. Age and Preference for Fusion Cuisine

**Figure 3:**
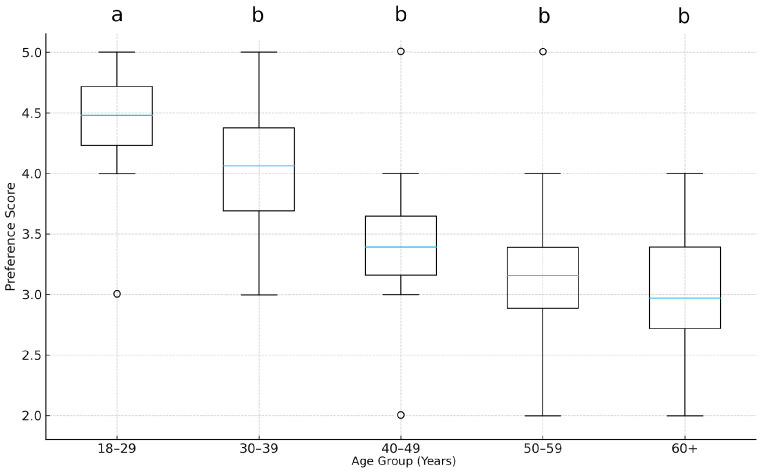
A boxplot of preference scores across age categories. Different letters above the boxplots indicate statistically significant differences among groups according to Dunn’s post hoc test (*p* < 0.05).

A Kruskal–Wallis test was conducted to examine whether preference scores varied significantly across age groups. The results showed a significant effect of age (H = 28.87, *p* < 0.001, η^2^_o_ = 0.09). As illustrated in Figure 3, younger participants (18–39 years) reported the highest median preference scores (median ≈ 4.5). In contrast, older participants (50+) exhibited lower scores (median ≈ 2.6). Dunn post hoc tests indicated that 18–39 > 40–49 (*p* < 0.01) and 18–39 > 50+ years (*p* < 0.01).

The results offer robust empirical support for Hypothesis 2 (H2), which posits that flavours combining culinary techniques from different nations are more appealing to younger than to older consumers. The findings highlight age as a significant determinant of openness toward gastronomic innovation and cross-cultural culinary experiences. Preference differed as a function of age group, H(4) = 28.87, *p* < 0.001, η^2^_o_ = 0.09 (see Figure 3). The finding of strong statistical significance (*p* < 0.001), combined with a small effect size (η^2^_o_ = 0.09), highlights that while age is a consistent factor shaping culinary openness, it does not operate in isolation but alongside other psychological and economic variables.

### 3.3. Income Level and Preference for Fusion Cuisine

**Figure 4:**
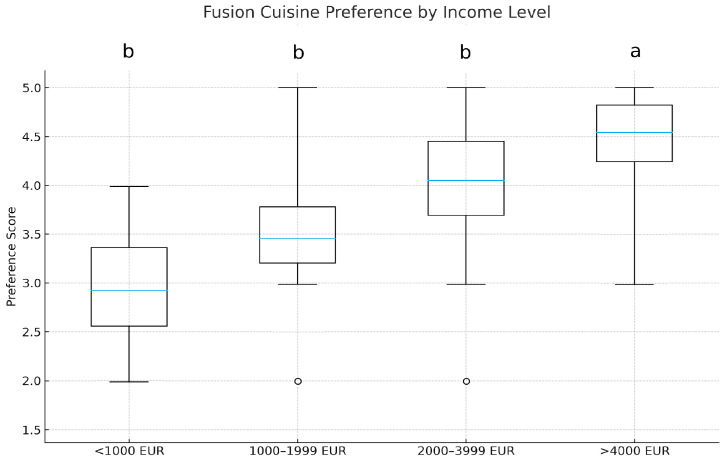
Preference scores by income level (in Euros). Different letters above the boxplots indicate statistically significant differences among groups according to Dunn’s post hoc test (*p* < 0.05).

A Kruskal–Wallis test was conducted to evaluate differences in fusion cuisine preference across income levels. A statistically significant effect was observed (H = 7.21, *p* = 0.042, η^2^_o_ = 0.02). As illustrated in Figure 4, participants in the “Very High Income” category (>4000€) exhibited the highest preference scores, whereas those in the “Low Income” group (<1000€) reported the lowest medians. Post hoc comparisons showed that >4000€ differed significantly from <1000€ (*p* < 0.05).

These findings provide strong empirical support for Hypothesis 3 (H3), according to which, those individuals having a higher income level exhibit a greater interest in foods and beverages reflecting culinary diversity. The results underscore the role of financial capacity in enabling access to and appreciation of gastronomic innovation. Preference varied by income, H(3) = 7.21, *p* = 0.042, η^2^_o_ = 0.02. (see Figure 4).

### 3.4. Religion and Fusion Cuisine Preference

**Figure 5:**
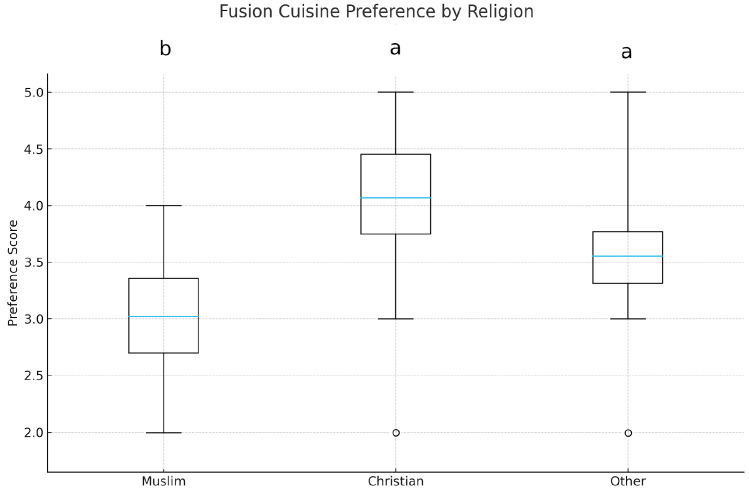
Variation in preference scores across religious groups. Different letters above the boxplots indicate statistically significant differences among groups according to Dunn’s post hoc test (*p* < 0.05).

A Kruskal–Wallis test was used to determine whether preference scores differed across religious affiliations. The analysis revealed a significant difference (H = 14.73, *p* = 0.001, η^2^_o_ = 0.05). As shown in Figure 5, Christians reported the highest median preference (≈4.0), followed by Other religions (≈3.5), and Muslims (≈3.0). Dunn post hoc tests showed the following: Christian > Muslim (*p* < 0.01), Christian > Other (n.s.), Other > Muslim (*p* < 0.05).

These findings indicate substantial variations in culinary openness among consumers of diverse religious affiliations. Initially, they endorse H4, indicating that Muslim participants had less favourable opinions towards meals that amalgamate components from different culinary traditions. Nonetheless, it is important to recognize that these discrepancies may arise not solely from religious identification, but also from the extent of restrictiveness of dietary regulations and compliance with these regulations. Research indicates that stringent dietary regulations, such as halal and kashrut, consistently influence consumer preferences and tend to diminish people’s receptiveness to unfamiliar foods [60,61,62]. The findings indicate that, alongside religious context, the perceived severity of dietary limitations and compliance with these constraints may significantly influence attitudinal variations in cross-cultural gastronomic preferences (preference differed by religion, H(4) = 14.73, *p* = 0.001, η^2^_o_ = 0.05 (see Figure 5). The statistical significance of the differences underscores the influence of religious context on gastronomic openness and cross-cultural culinary preferences.

### 3.5. Nationality and Preference for Fusion Cuisine

A Kruskal–Wallis test was conducted to assess whether preference scores differed across nationalities (Kosovo, Croatia, North Macedonia, Other). The analysis revealed no statistically significant differences (H = 3.19, *p* = 0.365, η^2^_o_ = 0.01). As illustrated in Figure 5, all groups showed similar distributions of preference scores.

**Figure 6:**
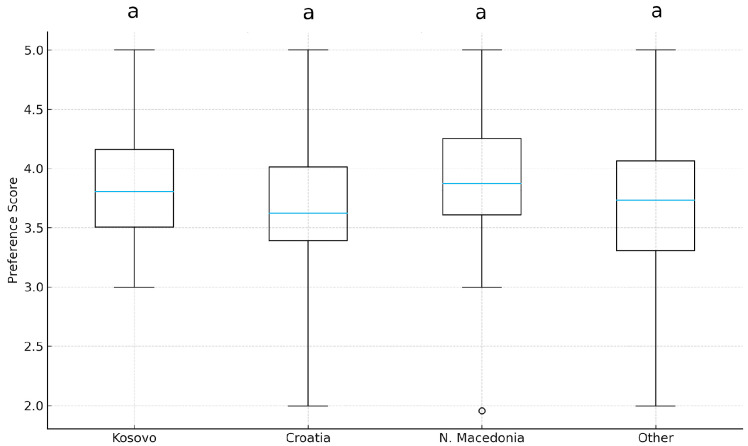
A boxplot of preference scores for different nationalities. Different letters above the boxplots indicate statistically significant differences among groups according to Dunn’s post hoc test (*p* < 0.05).

These findings indicate that Hypothesis 5 (H5), according to which consumers from Kosovo were assumed to exhibit a stronger preference for multicultural culinary experiences compared to other Balkan nationalities, is not supported by the data. Preference did not differ by nationality, H(3) = 3.19, *p* = 0.365 (see Figure 6).

### 3.6. Income Level and Culinary Diversity Interest Among Highly Educated Individuals

**Figure 7:**
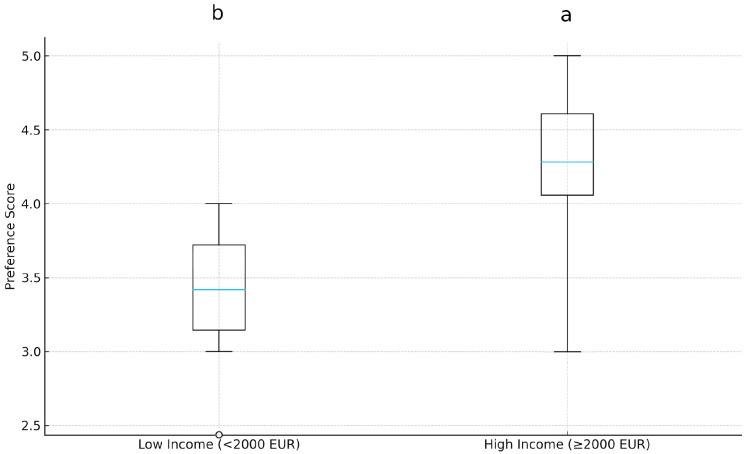
Interest scores by income level among highly educated participants. Different letters above the boxplots indicate a statistically significant difference between the two groups (Mann–Whitney U test, *p* < 0.05). Different letters above the boxplots indicate statistically significant differences among groups according to Dunn’s post hoc test (*p* < 0.05).

A Mann–Whitney U test was conducted to determine whether income influences culinary diversity interest among highly educated participants. The analysis revealed a significant difference (U = 5921, *p* = 0.041, Cliff’s δ = 0.24). Figure 7 shows that higher-income participants expressed greater interest in fusion dishes. It also shows that those in the High-Income group had higher average interest scores than consumers in the Low-Income group. This suggests that better educated people with higher incomes are more likely to be interested in trying new foods. Nonetheless, as emphasized in the literature [63,64], income level can significantly influence food choices and may specifically restrict access to more costly or specialized cuisines. Consequently, this disparity may stem not solely from cultural interests or personal preferences, but also from the significantly elevated cost of many fusion cuisine products, which diminishes accessibility for those individuals within the low-income demographic. These findings support Hypothesis 6 (H6), namely that income modulates gastronomic openness even within higher-educated demographics: high-income group > low-income group (U = 5921, *p* = 0.041, δ = 0.24).

These findings underscore the interaction between socioeconomic and educational factors in determining gastronomic preferences, reinforcing the idea that income level can modulate (Table 3).

## 4. Discussion

The findings of the present study indicate substantial correlations between individuals’ preferences for fusion cuisine and their educational attainment. In particular, those individuals with elevated educational attainment had a greater inclination towards fusion cuisine as compared to their less educated counterparts (H1). This corroborates other research indicating that educated consumers are more inclined to value the fusion of flavours and techniques from diverse cultures [1,43]. Nevertheless, it should be noted that the food cultures examined here share certain geographical and cultural proximities, which may render the fusion more familiar to participants than distant culinary combinations such as Peruvian–Japanese cuisine [65,66,67,68].

Conversely, those individuals with a lesser educational attainment would appear to exhibit a diminished interest in fusion cuisine, frequently preferring more accessible or familiar food options. This may occasionally encompass a taste for more traditional and culturally familiar flavours, but it may also incorporate fast food or other economical options [43,63]. Age was negatively associated with people’s preference for fusion cuisine (H2), aligning with the other findings [21,22,35], suggesting that flavours that result from combining different cooking techniques are more attractive to young consumers than older consumers.

This study also suggests a positive link between a preference for fusion cuisine and income levels (H3), aligning with research by Guiné et al. [16], and Luan and Kim [44], demonstrating that individuals with higher income levels may allocate more of their budget to gastronomic experiences from different culinary cultures. Consistent with the nonparametric findings, the observed differences across income groups were statistically significant but modest in magnitude, indicating that income contributes meaningfully but not exclusively to openness toward fusion cuisine.

However, it is important to emphasize that while these differences are statistically significant, the observed effect size was small (η^2^_o_ = 0.02). Therefore, statistical significance should not be interpreted as a strong practical effect, and income accounts for only a limited proportion of the variance in terms of people’s fusion cuisine preference.

Fusion cuisine preferences were also found to differ across religious groups (H4), consistent with prior research [47,48]. The results showed that Christians exhibited the highest preference for fusion cuisine, whereas Muslims had the lowest scores. However, given the ordinal nature of the data and the small-to-moderate effect sizes observed, these differences should be interpreted with caution. This difference could be due to halal dietary restrictions affecting Muslim consumers’ food choices, which makes them more careful regarding fusion cuisine. But this trend may also change depending on the main religion in a country. For example, Muslims who live in those countries where they are not the majority may be more open to fusion cuisine since they are more used to diverse cultures [1,47]. Conversely, Christians and consumers of other religions often exhibit more flexibility in their dietary preferences and may be more inclined to consume foods that blend elements from different culinary traditions due to less religious restrictions [48].

In contrast to the research of Vukovic and Terzic [53] and Rysha [52], this study assumed that Kosovo’s greater multiculturalism and food diversity would support a more positive view of culinary fusion than in other Balkan countries (H5). The expectation was that Kosovars would be more open to fusion cuisine. Yet, in line with the result of the Kruskal–Wallis test, no statistically significant difference was found between national groups, and the effect size was negligible. This suggests that national background may play a less influential role than individual-level demographic factors in shaping fusion cuisine preferences. Moreover, it is important to acknowledge that the absence of the expected differences in the Kosovars with the participants from other Balkan countries may be due to their relatively small sample size or their cultural similarity or similar culinary preferences with the other participants from Balkan countries examined in this study [54].

Those individuals with higher income levels exhibited significantly greater interest in culinary diversity than others (H6), consistent with prior research on socioeconomic differences in food choice and access to diverse culinary experiences [56,57]. This effect remained statistically significant in the subgroup analysis using the Mann–Whitney U test, although the magnitude (Cliff’s δ = 0.24) indicates a small effect. To reduce and document potential sample self-selection bias, data were collected during an open, public fusion cuisine event in Kosovo that was accessible to a broad range of visitors, rather than a closed, invitation-only setting. Participation occurred through on-site recruitment over the course of the event, and detailed demographic information (e.g., age, gender, education, income, nationality, religiosity) was recorded. The resulting sample showed substantial heterogeneity across these characteristics, suggesting that the data do not solely reflect a narrow, privileged subgroup but instead capture diverse consumer profiles [56,57,63]. Nevertheless, the voluntary nature of participation and the single-site design mean that some degree of self-selection cannot be completely ruled out and should be considered when generalizing the findings.

Given the unequal engagement patterns observed in this study, the present findings provide actionable design principles for more socially inclusive gastrodiplomatic initiatives. At the same time, the pattern observed for H6 provides actionable guidance for the design of future gastrodiplomatic initiatives. Prior studies indicate that individuals with higher levels of income and education are more exposed to gastronomic innovation, more willing to experiment with novel flavour combinations, and more appreciative of fusion cuisine and creative presentations [1,8,16,18,56,57]. Combined with the present results, this suggests that fusion-based gastrodiplomacy, if left entirely to market forces, is likely to disproportionately attract already advantaged, cosmopolitan publics. To counter this tendency, future programmes could deliberately locate fusion events in socioeconomically diverse neighbourhoods, reduce cost barriers (e.g., free tastings, subsidized menus), and incorporate educational components that explain the historical and cultural narratives behind Turkish–Kosovar fusion dishes [21,30,37,39,42]. Partnerships with schools, community centres, and cultural or tourism festivals may help embed such initiatives in everyday foodscapes rather than restricting them to exclusive venues [30,37,42]. In this way, the present findings do not merely document socioeconomic differences in the appreciation of fusion cuisine; they also point to concrete strategies for designing gastrodiplomatic activities that are more inclusive, culturally resonant, and socially balanced.

Additionally, Luša and Jakešević [41] suggested that the blended cultural influences in Balkan cuisines, such as Kosovo’s, may foster a greater interest in fusion cuisine. In addition, the research data were obtained during a gastrodiplomacy event. This enhances the practical value of the study by demonstrating how theoretical principles are reflected in real-world data. This is because research indicates that fusion food transcends personal preferences and serves as a mechanism for intercultural diplomacy [69] (p. 10).

Across the various hypotheses, the use of appropriate nonparametric effect sizes (ordinal η^2^ for Kruskal–Wallis tests and Cliff’s δ for the Mann–Whitney U test) demonstrate that demographic variables exert small-to-medium influences on fusion cuisine preference. This indicates that while demographic characteristics help shape individual openness to fusion cuisine, their influence is partial, and additional psychological or cultural factors likely play an important role. These demographic factors appear to shape openness to fusion cuisine, but, as might be expected, do not operate in isolation; their effects are modest when considered individually.

It is important to acknowledge that while psychological frameworks, such as sensation-seeking theory or food neophobia, may offer valuable insights into individuals’ engagement with fusion cuisine, these constructs fall outside the scope of this gastrodiplomacy-focused study. In particular, this study was designed to capture sensory responses and demographic patterns within a real-world cultural exchange setting, and the data collection procedures did not allow for assessment of broader psychological traits. Future work incorporating such theoretical perspectives would help deepen understanding of the mechanisms underlying individuals’ responses to fusion cuisine.

## 5. Conclusions

This study examined how individuals perceive the combination of flavours from different culinary traditions. Specifically, the study tested six hypotheses regarding the role of demographic variables, with the following outcomes:

Educational attainment (H1) and income level (H3) were positively associated with a preference for fusion cuisine. In addition, individuals with higher income levels demonstrated greater interest in culinary diversity (H6). Younger age was also confirmed as a significant predictor of openness to fusion flavours (H2). Preferences differed across religious groups (H4), with Christians scoring higher than Muslims. However, the observed effect sizes were small, suggesting that religious dietary considerations (e.g., halal-related concerns) may contribute to these differences but do not function as the sole determinant. Contrary to expectations, no statistically significant difference was found between Kosovar participants and those from other Balkan countries (H5), indicating that national background was less influential than individual-level socioeconomic factors in shaping fusion cuisine preferences within the examined context. Overall, the results indicate that socioeconomic status (education and income) and age are key drivers in shaping preferences for fusion cuisine. However, it is important to note that the effect sizes for these findings were generally small to moderate. Therefore, while demographics are significant, they do not fully explain the variance in consumer preferences.

Based directly on the finding that lower-education and lower-income groups are less responsive to fusion initiatives (H1, H3), this study suggests specific practical implications for gastrodiplomacy. To prevent gastrodiplomacy from remaining an elite activity, practitioners should consider integrating educational narratives into dining experiences—a strategy that directly addresses the “education gap” identified in our analysis. Furthermore, future research could investigate whether subsidized or community-based events effectively mitigate the income barriers observed in this study.

The findings reported here underline that cultural engagement through food encompasses cognitive and economic adaptation. Fusion cuisine appears to be an effective means of cultural communication, as the act of tasting food facilitates mutual recognition. The Kosovo–Türkiye case exemplifies how fusion cuisine, by integrating familiar and foreign elements, fosters dialogue, understanding, and mutual respect across historically connected yet distinct cultures.

This study contributes to the expanding corpus of information regarding the use of cuisine as an effective instrument for cultural diplomacy. It provides empirical evidence that deliberately organized culinary exchanges can improve cultural comprehension more effectively than symbolic or institutional efforts alone. Future research should aim to improve the bilateral aspect by integrating data from Türkiye, enabling a comparative analysis of people’s perception. Furthermore, qualitative techniques examining the sensory, emotional, and symbolic dimensions of tasting experiences should augment our comprehension of food as a medium for cultural diplomacy.

### Limitations

One limitation of this study is the absence of participants from Türkiye. This limitation reflects the contextual scope of the study, which was conducted exclusively in Kosovo as part of a unidirectional gastrodiplomatic effort. Future research could enhance the bilateral perspective by incorporating comparable data from Türkiye.

## Figures and Tables

**Table 1:** Distribution of Participants by Educational Attainment and Income Level.

	Category	n	Percentage (%)
**Educational Attainment**	Primary	50	11.1%
	Secondary	65	14.4%
	High School	110	24.4%
	Undergraduate	130	28.8%
	Postgraduate	96	21.3%
**Income Level (€/month)**	Low (<1000)	120	26.6%
	Medium (1000–1999)	150	33.3%
	High (2000–3999)	105	23.3%
	Very High (>4000)	76	16.8%
**Total**		451	100.0%

**Table 2:** Distribution of Participants by Nationality, Gender, and Age Group (in years).

Nationality	n	Female (%)	Male (%)			Age (%)		
				18–29	30–39	40–49	50–59	60+
Kosovo	78	44.9	55.1	26.9	23.1	19.2	17.9	12.9
Albania	70	45.7	54.3	25.7	24.3	18.6	20.0	11.4
Serbia	60	46.7	53.3	23.3	25.0	20.0	18.3	13.4
North Macedonia	56	42.9	57.1	21.4	26.8	19.6	17.9	14.3
Montenegro	42	47.6	52.4	19.0	23.8	21.4	21.4	14.3
Romania	35	48.6	51.4	22.9	25.7	20.0	20.0	11.4
Bulgaria	32	46.9	53.1	21.9	25.0	21.9	18.8	12.5
Bosnia and Herzegovina	30	43.3	56.7	23.3	26.7	20.0	16.7	13.3
Croatia	28	46.4	53.6	25.0	25.0	17.9	17.9	14.3
Other	20	50.0	50.0	20.0	25.0	25.0	15.0	15.0
Total	451	45.0	55.0	23.3	24.0	18.2	18.8	15.7

Note: Percentages are calculated within each nationality subgroup (N = 451).

**Table 3:** Hypothesis-wise inferential results (H1–H6) in the full sample (N = 451): tests, degrees of freedom, test statistics, *p*-values, and effect sizes.

Hypothesis	Test	df	Statistic	*p*-Value	Effect Size
H1 (Education)	Kruskal–Wallis	4	H = 32.41	<0.001	η^2^_o_ = 0.11
H2 (Age)	Kruskal–Wallis	2	H = 28.87	<0.001	η^2^_o_ = 0.09
H3 (Income)	Kruskal–Wallis	3	H = 7.21	0.042	η^2^_o_ = 0.02
H4 (Religion)	Kruskal–Wallis	2	H = 14.73	0.001	η^2^_o_ = 0.05
H5 (Nationality)	Kruskal–Wallis	3	H = 3.19	0.365	η^2^_o_ = 0.01
H6 (Income × Education)	Mann–Whitney U	-	U = 5921	0.041	Cliff’s δ = 0.24

## Data Availability

The original contributions presented in the study are included in the article. Further inquiries can be directed to the corresponding author.

## Appendix A

**Füzyon Mutfak: Kosova&Türkiye**

Sizi “Füzyon Mutfak: Kosova&Türkiye Karşılaştırması” başlıklı araştırmaya davet ediyoruz. Bu araştırmaya katılıp katılmama kararını vermeden önce, araştırmanın neden ve nasıl yapılacağını bilmeniz gerekmektedir. Bu nedenle bu formun okunup anlaşılması büyük önem taşımaktadır. Eğer anlayamadığınız ve sizin için açık olmayan şeyler varsa, ya da daha fazla bilgi isterseniz bize sorunuz.

Bu çalışmaya katılmak tamamen gönüllülük esasına dayanmaktadır. Çalışmaya katılmama veya katıldıktan sonra herhangi bir anda çalışmadan çıkma hakkında sahipsiniz. Çalışmayı yanıtlamanız, araştırmaya katılım için onam verdiğiniz biçiminde yorumlanacaktır. Size verilen formlardaki soruları yanıtlarken kimsenin baskısı veya telkini altında olmayın. Bu formlardan elde edilecek bilgiler tamamen araştırma amacı ile kullanılacaktır. Dr. Ceyhun UÇUK (ceyhunucuk@gmail.com), Gaziantep Üniversitesi

**Kuzhina Fusion: Kosovë&Turqi**

Ju ftojmë në hulumtimin me titull “Kuzhina Fusion: Krahasimi Kosovë&Turqi”. Përpara se të vendosni nëse do të merrni pjesë në këtë hulumtim apo jo, është e nevojshme të dini pse dhe si do të bëhet ky hulumtim. Prandaj, është shumë e rëndësishme që ky formular të lexohet dhe të kuptohet. Nëse ka gjëra që nuk i kuptoni ose që nuk janë të qarta për ju, ose nëse dëshironi më shumë informacion, ju lutemi na pyesni.

Pjesëmarrja në këtë studim është plotësisht vullnetare. Ju keni të drejtë të mos merrni pjesë në studim ose të largoheni nga studimi në çdo moment pas pjesëmarrjes. Përgjigjja juaj ndaj studimit do të interpretohet si dhënie e pëlqimit për pjesëmarrje në hulumtim. Mos lejoni që të jeni nën presion ose sugjerim nga dikush tjetër kur përgjigjeni pyetjeve në formularët e dhënë. Informacioni i marrë nga këto formularë do të përdoret vetëm për qëllime kërkimore. Dr. Ceyhun UÇUK (ceyhunucuk@gmail.com), Universiteti i Gaziantep

**Fusion Cuisine: Kosovo & Türkiye**

You are invited to take part in a research study titled “Fusion Cuisine: A Comparative Study between Kosovo & Türkiye.” Before deciding whether to take part in this study, it is important that you understand why the research is being conducted and how it will be carried out. Therefore, carefully reading and fully understanding this form is highly important. If there is anything unclear or if you would like more information, please do not hesitate to ask us.

Participation in this study is entirely voluntary. You have the right to decline participation or to withdraw from the study at any time, even after you have agreed to take part. Completing the survey will be considered as your consent to participate in the study. While answering the questions provided, please ensure that you are not influenced or pressured by anyone. The information collected from these forms will be used solely for research purposes. Dr. Ceyhun UÇUK (ceyhunucuk@gmail.com), University of Gaziantep

1. **Yaşınız (Mosha juaj, Age in years) ***

18–29
30–39
40–49
50–59
60 ve üzeri (58 dhe mbi/60 and above)

2. **Cinsiyetiniz (Gjinia juaj/Gender) ***

Kadın (Grua/Female)
Erkek (Burrë/Male)
Belirtmek İstemiyorum (Nuk dua të tregoj/Prefer not to say)

3. **En Son Mezun Olunan Derece (Shkalla e fundit e përfunduar/ Highest level of education completed) ***

İlkokul (Shkolla fillore/Primary School)
Ortaokul (Shkolla e mesme e ulët/Lower secondary school-Middle school)
Lise (Shkolla e mesme e lartë/High School)
Önlisans (Diplomë e shkollës së lartë/Associate Degree)
Lisans (Diplomë Bachelor/Bachelor’s Degree)
Yüksek Lisans (Master/Master’s Degree)
Doktora (Doktoraturë/Doctorate-PhD)

4. **Medeni Durum (Statusi martesor Marital Status) ***

Evli (I martuar/married)
Bekar (Beqar/single)

5. **Meslek** **(Profesioni/Occupation-Profession) ***

6. **Aylık Geliriniz (Të ardhurat tuaja mujore/Monthly income) ***

<1000 EUR
1000–1999 EUR
2000–3999 EUR
≥4000 EUR

7. **Hangi Ülkede İkamet Ediyorsunuz? (Në cilin vend banoni/Which country do you reside in?) ***

8. **Etnik Kökeniniz Nedir? (Cili është origjina juaj etnike?/What is your ethnic origin?) ***

9. **Hangi Dine Mensupsunuz? (Cilit besim i përkisni?/What is your religion?) ***

Hristiyan (I krishterë/Christian)
Müslüman (Mysliman/Muslim)
Diğer (Tjetër/Other)

10. **Bir tabakta farklı ulusların malzemelerini birleştiren lezzetleri tatmayı tercih ederim. (Preferoj të shijoj shijet që kombinojnë përbërësit e kombeve të ndryshme në një pjatë/I prefer to taste flavours that combine ingredients from different national cuisines in a single dish) ***

Kesinlikle Katılmıyorum (Nuk jam absolutisht dakord/Strongly Disagree)
Katılmıyorum (Nuk jam dakord/Disagree)
Ne Katılıyorum Ne Katılmıyorum (As jam dakord, as nuk jam dakord/Neither Agree nor Disagree)
Katılıyorum (Jam dakord/Agree)
Kesinlikle Katılıyorum (Jam absolutisht dakord/Strongly Agree)

11. **Bir tabakta farklı ulusların pişirme tekniklerini birleştiren lezzetleri tatmayı tercih ederim. (Preferoj të shijoj shijet që kombinojnë teknikat e gatimit të kombeve të ndryshme në një pjatë/I prefer to taste flavours that combine cooking techniques from different national cuisines in a single dish) ***

Kesinlikle Katılmıyorum (Nuk jam absolutisht dakord/Strongly Disagree)
Katılmıyorum (Nuk jam dakord/Disagree)
Ne Katılıyorum Ne Katılmıyorum (As jam dakord, as nuk jam dakord/Neither Agree nor Disagree)
Katılıyorum (Jam dakord/Agree)
Kesinlikle Katılıyorum (Jam absolutisht dakord/Strongly Agree)

12. **Farklı mutfak kültürlerinin birleşiminden oluşan, ancak birinin diğerine baskın olmadığı gıdaları denemekten hoşlanırım. (Më pëlqen të provoj ushqime që përbëhen nga kombinimi i kulturave të ndryshme të kuzhinës, por ku asnjëra prej tyre nuk dominon mbi tjetrën/I enjoy trying foods created by the combination of different culinary cultures, where none is dominant over the other) ***

Kesinlikle Katılmıyorum (Nuk jam absolutisht dakord/Strongly Disagree)
Katılmıyorum (Nuk jam dakord/Disagree)
Ne Katılıyorum Ne Katılmıyorum (As jam dakord, as nuk jam dakord/Neither Agree nor Disagree)
Katılıyorum (Jam dakord/Agree)
Kesinlikle Katılıyorum (Jam absolutisht dakord/Strongly Agree)

13. **Farklı kültürlere ait yiyecek ve içecek malzemelerinin bir araya getirilerek sıra dışı bir şekilde sunulan yiyecek ve içecekler ilgilimi çeker. (Jam i interesuar për ushqimet dhe pijet që përbëhen nga përbërës të ndryshëm nga kultura të ndryshme dhe që ofrohen në një mënyrë të pazakontë/ I am interested in food and beverages that creatively combine ingredients from different cultures and present them in an unusual way) ***

Kesinlikle Katılmıyorum (Nuk jam absolutisht dakord/Strongly Disagree)
Katılmıyorum (Nuk jam dakord/Disagree)
Ne Katılıyorum Ne Katılmıyorum (As jam dakord, as nuk jam dakord/Neither Agree nor Disagree)
Katılıyorum (Jam dakord/Agree)
Kesinlikle Katılıyorum (Jam absolutisht dakord/Strongly Agree)

14. **Farklı kültürlere ait yiyecek ve içecek malzemelerinin bir araya getirilmesi ile hazırlanan sıra dışı tatlara sahip olan yiyecek ve içecekler ilgilimi çeker. (Jam i interesuar për ushqime dhe pije që kanë shije të pazakonta dhe që përgatiten duke kombinuar përbërës nga kultura tëndryshme/ I am interested in food and beverages that have unusual flavours as a result of combining ingredients from different cultures) ***

Kesinlikle Katılmıyorum (Nuk jam absolutisht dakord/Strongly Disagree)
Katılmıyorum (Nuk jam dakord/Disagree)
Ne Katılıyorum Ne Katılmıyorum (As jam dakord, as nuk jam dakord/Neither Agree nor Disagree)
Katılıyorum (Jam dakord/Agree)
Kesinlikle Katılıyorum (Jam absolutisht dakord/Strongly Agree)

15. **Farklı kültürlere ait yiyecek ve içecek malzemelerinin bir araya getirilmesi ile hazırlanan sıra dışı kokulara sahip olan yiyecek ve içecekler ilgilimi çeker. (Jam i interesuar për ushqime dhe pije që kanë aroma të pazakonta dhe që përgatiten duke kombinuar përbërës nga kultura tëndryshme/I am interested in food and beverages that have unusual aromas created by combining ingredients from different cultures) ***

Kesinlikle Katılmıyorum (Nuk jam absolutisht dakord/Strongly Disagree)
Katılmıyorum (Nuk jam dakord/Disagree)
Ne Katılıyorum Ne Katılmıyorum (As jam dakord, as nuk jam dakord/Neither Agree nor Disagree)
Katılıyorum (Jam dakord/Agree)
Kesinlikle Katılıyorum (Jam absolutisht dakord/Strongly Agree)

16. **Farklı kültürlere ait yiyecek ve içecek malzemelerinin bir araya getirilmesi ile hazırlanan özgün yiyecek ve içecekler ilgimi çeker. (Jam i interesuar për ushqime dhe pije unike që përgatiten duke kombinuar përbërës nga kultura të ndryshme/I am interested in novel foods and beverages created by combining ingredients from different cultures) ***

Kesinlikle Katılmıyorum (Nuk jam absolutisht dakord/Strongly Disagree)
Katılmıyorum (Nuk jam dakord/Disagree)
Ne Katılıyorum Ne Katılmıyorum (As jam dakord, as nuk jam dakord/Neither Agree nor Disagree)
Katılıyorum (Jam dakord/Agree)
Kesinlikle Katılıyorum (Jam absolutisht dakord/Strongly Agree)

17. **Farklı mutfak kültürlerine ait pişirme teknikleri bir araya getirilerek sıra dışı bir şekilde sunulan yiyecek ve içecekler ilgilimi çeker. (Jam i interesuar për ushqime dhe pije që ofrohen në një mënyrë të pazakontë duke kombinuar teknika gatimi nga kultura të ndryshme të kuzhinës/I am interested in food and beverages that are presented in an unusual way by combining cooking techniques from different culinary cultures) ***

Kesinlikle Katılmıyorum (Nuk jam absolutisht dakord/Strongly Disagree)
Katılmıyorum (Nuk jam dakord/Disagree)
Ne Katılıyorum Ne Katılmıyorum (As jam dakord, as nuk jam dakord/Neither Agree nor Disagree)
Katılıyorum (Jam dakord/Agree)
Kesinlikle Katılıyorum (Jam absolutisht dakord/Strongly Agree)

18. **Farklı mutfak kültürlerine ait pişirme tekniklerinin bir araya getirilmesi ile hazırlanan sıra dışı tatlara sahip olan yiyecek ve içecekler ilgilimi çeker. (Jam i interesuar për ushqime dhe pije që kanë shije të pazakonta dhe që përgatiten duke kombinuar teknika gatimi nga kultura të ndryshme të kuzhinës/ I am interested in food and beverages that have unusual flavours created by combining cooking techniques from different culinary cultures) ***

Kesinlikle Katılmıyorum (Nuk jam absolutisht dakord/Strongly Disagree)
Katılmıyorum (Nuk jam dakord/Disagree)
Ne Katılıyorum Ne Katılmıyorum (As jam dakord, as nuk jam dakord/Neither Agree nor Disagree)
Katılıyorum (Jam dakord/Agree)
Kesinlikle Katılıyorum (Jam absolutisht dakord/Strongly Agree)

19. **Farklı mutfak kültürlerine ait pişirme tekniklerinin bir araya getirilmesi ile hazırlanan sıra dışı kokulara sahip olan yiyecek ve içecekler ilgilimi çeker. (Jam i interesuar për ushqime dhe pije që kanë aroma të pazakonta dhe që përgatiten duke kombinuar teknika gatimi nga kultura të ndryshme të kuzhinës/I am interested in food and beverages that have unusual aromas created by combining cooking techniques from different culinary cultures) ***

Kesinlikle Katılmıyorum (Nuk jam absolutisht dakord/Strongly Disagree)
Katılmıyorum (Nuk jam dakord/Disagree)
Ne Katılıyorum Ne Katılmıyorum (As jam dakord, as nuk jam dakord/Neither Agree nor Disagree)
Katılıyorum (Jam dakord/Agree)
Kesinlikle Katılıyorum (Jam absolutisht dakord/Strongly Agree)

20. **Farklı mutfak kültürlerine ait pişirme tekniklerinin bir araya getirilmesi ile hazırlanan özgün yiyecek ve içecekler ilgilimi çeker. (Jam i interesuar për ushqime dhe pije unike që përgatiten duke kombinuar teknika gatimi nga kultura të ndryshme të kuzhinës/ I am interested in unique foods and beverages that are created by combining cooking techniques from different culinary traditions.)**

Kesinlikle Katılmıyorum (Nuk jam absolutisht dakord/Strongly Disagree)
Katılmıyorum (Nuk jam dakord/Disagree)
Ne Katılıyorum Ne Katılmıyorum (As jam dakord, as nuk jam dakord/Neither Agree nor Disagree)
Katılıyorum (Jam dakord/Agree)
Kesinlikle Katılıyorum (Jam absolutisht dakord/Strongly Agree)

## Appendix B

	**Food Name:** Krempita & Baklava	**About:** This recipe combines Krempita, a traditional layered cream pastry popular in the Balkans, and Baklava, a symbolic dessert of Turkish cuisine. Designed as a fusion dessert, it represents the gastronomic and cultural connection between Türkiye and Kosovo, offering high sensory richness through creamy texture and pistachio aroma.		
	**Total Time:** 4 h			
	**Pax:** 10			
	**Ingredients**			
	**For Cream Layer** - 2000 mL Milk - 300 g Granulated sugar - 8 pcs Egg yolks - 200 g Cornstarch - 4 packs (32 g total) Vanilla (powder) - Butter: 200 g - Cream (35% fat): 1000 mL		**For Baklava Sheet Layer** - 20 sheets (size 40 × 40 cm) Baklava phyllo dough - 500 g Ground pistachio - 250 g Clarified butter for brushing **For Syrup** - 300 g Granulated sugar - 200 mL Water - 10 mL Lemon juice	
				***Mise en Place* Procedure:** - Scale all ingredients using precision scale - Keep milk and cream at +4 °C - Clarify butter and keep at 50–55 °C during use - Keep phyllo sheets covered to prevent drying - Preheat oven to 185 °C (fan off) - Prepare deep stainless steel baking tray (32 × 20 cm)
	**Requirements (Tools & Equipment)**			
	- Stainless steel saucepan (5 L, thick-bottomed) - 32 × 20 cm steel baking tray - Food thermometer (0–100 °C) - Hand whisk + silicone spatula		- Precision scale (0.1 g accuracy) - Rapid cooling fridge (+4 °C) - Fine strainer	
**Stage**	**Procedure**			
1	Heat milk and 150 g sugar at 90–95 °C until dissolved			
2	Whisk egg yolks, remaining sugar, cornstarch, and vanilla until smooth			
3	Temper hot milk into yolk mixture slowly			
4	Return to heat, cook until cream reaches 83–85 °C and thickens			
5	Remove from heat, add butter, cool rapidly to 25–30 °C			
6	Fold cream with cold heavy cream to achieve aerated structure			
7	Brush phyllo sheets individually with butter, layer 10 sheets in tray			
8	Spread half pistachio			
9	Add remaining 10 sheets, finish with butter brushing			
10	Bake at 185 °C for 24 min until golden			
11	Prepare hot syrup: boil 300 g sugar + 200 mL water for 6 min, add lemon			
12	Pour hot syrup over baked layers, rest 60 min			
13	Spread cream layer evenly (3–4 cm thickness)			
14	Chill in blast chiller +4 °C for min. 120 min			
15	Portion as 8 × 8 cm squares for sensory testing			

	** Food Name: ** Ajvar & Haydari Canapés	**About:** Ajvar, a Balkan-origin roasted pepper-eggplant paste, and Haydari, a traditional Turkish strained yoghurt dip, are combined on toasted baguette slices to create a fusion cold starter. This appetizer represents Kosovo–Türkiye culinary diplomacy through complementary flavour profiles and shared meze culture.		
	**Total Time:** 2 h			
	**Pax:** 10			
	**Ingredients**			
	**For Ajvar** - 2000 g Red bell peppers - 1000 g Eggplant - 100 g Garlic - 150 mL Olive oil - 25 mL Grape vinegar - 25 g Salt - 10 g Black pepper		**For Haydari** - 500 g Strained yoghurt - 200 g White cheese (crumbled) - 30 g Dill - 20 g Fresh mint - 20 mL Olive oil - 5 g Salt - 10 g Garlic **For Service** - 3 pcs Baguette bread - 150 g Cucumber (thin slices)	
				***Mise en Place* Procedure:** - Preheat oven to 220 °C - Wash all vegetables under running potable water - Chill yoghurt at +4 °C - Prepare cutting board + chef’s knife for fine chopping - Slice baguette at 1.5 cm thickness, place on baking tray - Set blast chiller to +4 °C for rapid cooling of roasted vegetables
	**Requirements (Tools & Equipments)**			
	- Fan-assisted oven (220 °C) - 30 × 40 cm baking tray - Hand blender (two-stage recommended)		- Fine strainer/sieve - Mixing bowls (2–3 L) - Food safety gloves - Instant-read thermometer	
**Stage**	**Procedure**			
1	Roast peppers & eggplants at 220 °C for 25–30 min until charred			
2	Transfer to covered container for 10 min → steam → easy peeling			
3	Peel skins, remove seeds, drain excess water			
4	Blend roasted vegetables, garlic, salt, pepper, vinegar			
5	Add olive oil gradually to emulsify Ajvar structure			
6	Chill Ajvar to 10 °C before plating			
7	Combine strained yoghurt, white cheese, finely chopped dill & mint			
8	Add olive oil, garlic, salt → mix until smooth			
9	Toast bread slices at 180 °C for 6–7 min			
10	Spread Haydari (10–12 g each), top with Ajvar (20–25 g)			
11	Garnish with cucumber slice			
12	Serve immediately at 10–12 °C			

	** Food Name: ** Flija & Sac Tava	**About:** Flija is a layered baked batter dish carrying strong cultural value in Kosovo and for broader Balkan culinary heritage. Sac Tava, on the other hand, is a traditional Turkish main course in which meat and vegetables are cooked over high heat to achieve a rich savoury profile. The two dishes are combined into a single plate format, representing a fusion interpretation of the shared gastronomic heritage of Türkiye and Kosovo.		****
	**Total Time:** 6			
	**Pax:** 10			
	**Ingredients**			
	**For Flija Batter** - 2000 g Flour - 2000 mL Milk - 12 Eggs - 500 mL Water - 500 g Yoghurt - 30 g Salt - 300 g Butter (for brushing)		**For Sac Tava** - Diced lamb or beef: 5000 g - Onion: 1000 g - Green & red peppers: 1000 g - Tomatoes: 1000 g - Garlic: 10 cloves (25 g) - Olive oil: 300 mL - Butter: 150 g - Dried thyme: 50 g - Chilli flakes: 50 g - Black pepper: 50 g - 50 g Salt	
				***Mise en Place* Procedure:** - Set sac lid heating system to 250–300 °C - Dice meat into 2 × 2 cm cubes - Chop vegetables into 1.5 cm pieces - Keep proteins at +4 °C until needed - Prepare steel baking tray (38 × 28 cm) - Food contact areas sanitized (HACCP compliance)
	**Requirements (Tools & Equipments)**			
	- Traditional sac lid (upper heating source) - Stainless steel tray 38 × 28 cm - Thick-bottomed sauté pan (20 mm)		- Hand whisk - Silicone brush - Blast refrigerator (+4 °C) - Food thermometer (up to 300 °C)	
**Stage**	**Procedure**			
1	Combine flour, milk, eggs, yoghurt, salt → whisk until homogeneous			
2	Pour a thin layer of batter into tray			
3	Brush lightly with melted butter			
4	Cook under heated sac lid for 5–7 min			
5	Repeat steps 2–4 for 15–18 layers total			
6	After final layer, cool to 40 °C			
7	Heat sauté pan with olive oil at 200 °C			
8	Brown diced meat on high heat: 5–7 min			
9	Add onions, peppers, tomatoes, garlic → sauté 10 min			
10	Add spices & butter → simmer 45–60 min at 95–98 °C			
11	Plate Sac Tava over sliced Flija squares (10 × 10 cm each)			
12	Rest dish at 60–65 °C during service window			
